# Examining the Effects of Social Media on Mental Health Among Adolescents in Saudi Arabia

**DOI:** 10.7759/cureus.53261

**Published:** 2024-01-30

**Authors:** Fahad Ali Faqihi, Rayan A Qutob, Ruba Hamed Mohammed Subh, Lama Abdullah Mohammed Aljathalin, Lina Ziyad Alshalan, Sara Mohammed Alhanshi Yati, Abdullah Alaryni, Abdullah Alghamdi, Eysa Alsolamy, Abdullah Bukhari, Abdulrahman Alanazi, Bayan Hussain Abdullah Hazazi, Haifa Abdulrahman Abuhemid, Reema Abdulrahman Alassaf, Sarah Saad Muidh Alzahrani

**Affiliations:** 1 Department of Internal Medicine and Adult Critical Care Medicine, Dr. Sulaiman Al Habib Medical Group Holding Company, Riyadh, SAU; 2 Department of Internal Medicine, College of Medicine, Imam Mohammad Ibn Saud Islamic University, Riyadh, SAU; 3 College of Medicine, Imam Mohammad Ibn Saud Islamic University, Riyadh, SAU

**Keywords:** psychology, saudi arabia, adolescents, mental, social media

## Abstract

Background: Adolescents are immersed in a highly advanced technology setting, characterized by extensive availability of personal devices, the internet, and other social media platforms, which greatly impact their mental development. Concerns arise around potential negative consequences, including social isolation, internet addiction, exposure to improper content, depression, cyberbullying, and sleeping difficulty. The impact of social media on the mental well-being of adolescents has emerged as an escalating concern. The continuous exposure to carefully selected content, online harassment, and the expectation to comply with unrealistic standards all contribute to the development of depression and anxiety. This research aims to explore the impact of social media on the mental health of adolescents in the Saudi Arabian context.

Methods: This online cross-sectional study was conducted on Saudis aged 10 to 24 years old who have social media accounts in Saudi Arabia between August and November 2023. Binary logistic regression analysis was conducted to identify factors that make the study participants more likely to be mentally affected by social media.

Results: A total of 2856 participants were involved in this study. The most commonly used social media platform by the study participants was Twitter (30.3%). The majority of the study participants (77.4%) reported that they have tried to reduce their social media usage for mental health reasons. The most commonly agreed-upon statements were that “they feel that excessive social media use has negatively affected their sleep patterns,” “they often sleep late in the night because of social media usage,” and “they check their social media account before they sleep in the night,” accounting for 71.0%, 66.1%, and 58.3%, respectively. Overall, the study participants showed a low level of being mentally affected due to social media with a mean score of 7.8 (SD: 3.9) out of 21, which is equal to 37.1%. Older participants (aged 16-24 years) were more prone to be mentally affected due to social media compared to younger ones (aged 10-15 years; p < 0.05). On the other hand, participants who were married, had a middle school education level, or lived in the northern, western, or eastern regions were less likely to be mentally affected due to social media compared to others (p < 0.05).

Conclusion: The study participants showed a low level of being mentally affected due to social media. Individuals in the 16-24 years age group show a higher susceptibility to mental impacts resulting from social media usage. The primary duty for reducing the potential negative effects of social media should not rest only on parents and caregivers. Advocating for the creation of technology-free zones and fostering in-person friendships among teens could be a beneficial approach that policymakers should support.

## Introduction

In today's digital age, adolescents are engaged in a technologically advanced environment marked by widespread access to personal devices, the internet, and various social media platforms, significantly influencing their psychosocial development [1,2]. This increase in digital engagement presents both benefits and concerns. Adolescents benefit from international networking, the development of information and communications technology (ICT) skills, information access, self-exploration, and self-expression [3-5]. However, concerns arise regarding negative consequences such as social isolation, internet addiction, exposure to inappropriate content, depression, cyberbullying, and sleep deprivation [1-3].

As the influence of social media in our daily lives continues to expand significantly [6,7], adolescents are committing an escalating amount of their time to diverse online networking platforms, participating in networking, e-games, and messaging [8,9]. Prior studies suggest a correlation between heightened social media use and an increase in mental health problems [5,10-12]. Maintaining good mental health is integral to the overall well-being of individuals, including adolescents [13]. The World Health Organization (WHO) [14] notes that mental health issues arise in late childhood and early adolescence, affecting about 10% of children and adolescents globally. Besides, a previous study found that generalized anxiety disorder and depression are most prevalent among children and adolescents [15].

In 2023, Saudi Arabia recorded a substantial social media usage rate of 79.3%, encompassing approximately 29.1 million individuals [16]. Within the Saudi population, a notable percentage of young individuals engage with social media [17]. Saudis attribute their use of social media to staying connected with friends and family, utilizing free time, and exploring products for purchase, ranking these as the top three reasons [18]. Notably, mental health issues in the Kingdom of Saudi Arabia (KSA) are estimated to affect around 20.2% of the population [19].

Understanding the intricate association between social media usage and the mental health of adolescents is crucial to navigating the complexities of this digital era. This research aims to explore the impact of social media on the mental health of adolescents, specifically in the Saudi Arabian context.

## Materials and methods

Study design and setting

This is an online cross-sectional study that was conducted on Saudis aged 10-24 years who have social media accounts in Saudi Arabia between August and November 2023.

Study population

The study included individuals aged 10-24 years from several places across Saudi Arabia. All participants must satisfy the following requirements: they must be residents of Saudi Arabia of both genders, living in any region of Saudi Arabia. Additionally, their age must fall between the range of 10 and 24 years. The individuals who declined to participate in this study were excluded.

Sampling technique

This study utilized a convenient sampling strategy. This study was conducted using an electronic self-administered questionnaire that was distributed through social media (Facebook, Twitter, Instagram, and WhatsApp), which was distributed randomly to determine the effects of social media on mental health among adolescents in Saudi Arabia.

Questionnaire tool

The questionnaire tool was developed based on an extensive literature review in the area of the effects of social media on mental health among adolescents. The questionnaire contained two sections: demographics (age group, gender, nationality, marital status, education, employment, and region of residency) and measuring the impact of social media on mental health. The mental impact of social media was examined using 21 items. The confirmation given to the participant for each item gave him/her a score of one, for a total score of 21. The higher the score, the more mentally affected the participants will be.

Questionnaire piloting

The questionnaire instrument was evaluated and confirmed by two researchers. They were surveyed regarding the readability, comprehensibility, and overall validity of the questions. Additionally, they were inquired about their perception of any questions that they considered disagreeable. They stated that completing the questionnaire was straightforward. The face validity of the items was confirmed, assessing their clarity, comprehensibility, relevance, and suitability of response alternatives. In addition, they verified the suitability of the survey's duration, the time it took to complete, and the relevance of the scale used. Prior to administering the questionnaire to the entire community, researchers ran a pilot study to assess the level of interest and understanding. In the pilot phase, a sample of 25 participants was randomly selected from different regions of Saudi Arabia to complete the questionnaire. The results of the primary investigation did not incorporate the responses provided by these participants.

Sample size

The required sample size from each study population was 385 participants, based on a confidence interval of 95%, a standard deviation of 0.5, and a margin of error of 5%.

Ethical approval

The ethical approval for this study was obtained from the ethical research committee of the Institutional Review Board (IRB) of Imam Mohammed Ibn Saud Islamic University (IMSIU), Riyadh, Saudi Arabia, wherein they reviewed and approved this project (HAPO-01-R-0011; Project No. 517/2023). Informed consent was obtained from all participants. Comprehensive information regarding the study was provided to participants and parents (for those aged 10-17 years), thereby facilitating understanding and enabling them to freely and carefully choose to participate. The families and respondents were formally notified that their completion of the questionnaire would be regarded as their providing written informed consent.

Statistical analysis

The data were analyzed using SPSS version 28 (IBM Corp., Armonk, NY). Categorical variables were represented using numerical values and percentages (%), whereas continuous variables were displayed as the mean and standard deviation (SD). The normality assessment was conducted utilizing the Shapiro-Wilk test and the Kolmogorov-Smirnov test. The study employed binary logistic regression analysis to identify factors that make the study participants more likely to be mentally affected by social media. The binary logistic regression analysis employed a dummy variable, which was determined based on the mean score of items that examined the mental impact of social media. The statistical significance level was set at 5.0%.

## Results

Participants' demographic characteristics

A total of 2856 participants were involved in this study. More than half of the study participants (53.9%) were aged 21-24 years. The majority of the study participants (77.0%) were female. The vast majority of the study participants (91.7%) were Saudis. The majority of the study participants (79.6%) were single. Around 47.2% of them reported that they have a university degree. Around one-third of the study participants (31.3%) reported that they live in the western region. For further details on the participants' demographic characteristics, refer to Table 1.

**Table 1:** Participants' demographic characteristics

Variable	Frequency	Percentage
Age group		
10-15 years	241	8.4%
16-20 years	1076	37.7%
21-24 years	1539	53.9%
Gender		
Females	2200	77.0%
Nationality		
Saudi	2618	91.7%
Marital status		
Single	2273	79.6%
Married	524	18.3%
Divorced	46	1.6%
Widowed	13	0.5%
Education		
Primary school or lower	66	2.3%
Middle school	160	5.6%
High school	1021	35.7%
Diploma	201	7.0%
University degree	1349	47.2%
Higher education	59	2.1%
Employment		
Student	1935	67.8%
Employed	470	16.5%
Unemployed	451	15.8%
Region of residency		
Central region	540	18.9%
Northern region	465	16.3%
Southern region	622	21.8%
Western region	894	31.3%
Eastern region	335	11.7%

Social media utilization profile

Table 2 below presents participants’ social media utilization profile. The most commonly used social media platform by the study participants was Twitter (30.3%). More than half of the study participants (52.5%) reported that they started using social media before the age of 10. Around 37.7% of the study participants reported that they use social media platforms for four to six hours per day. The vast majority of the study participants (85.2%) reported that they are aware of mental health resources and support available through social media platforms. The majority of the study participants (77.4%) reported that they have tried to reduce their social media usage for mental health reasons. Around 69.9% of the study participants reported that they are comfortable (neutral, comfortable, or very comfortable) seeking professional help for mental health issues related to social media use. The vast majority of the study participants (94.5%) reported that they are aware of privacy settings and the importance of protecting personal information on social media.

**Table 2:** Social media utilization profile

Variable	Frequency	Percentage
Which social media platforms do you use the most? (Select all that apply)		
Twitter	866	30.3%
Snapchat	850	29.8%
Facebook	617	21.6%
Instagram	467	16.4%
TikTok	56	2.0%
At which age you started using social media?		
Before 10 years of age	1500	52.5%
10-15 years	736	25.8%
16-20 years	319	11.2%
21-24 years	301	10.5%
How frequently do you use social media platforms?		
Rarely	63	2.2%
Less than an hour per day	126	4.4%
1-3 hours per day	691	24.2%
4-6 hours per day	1077	37.7%
More than 6 hours per day	899	31.5%
Are you aware of mental health resources and support available through social media platforms?		
Yes	2432	85.2%
Have you ever taken breaks or tried to reduce your social media usage for mental health reasons?		
Yes	2210	77.4%
How comfortable do you feel seeking professional help for mental health issues related to social media use?		
Very uncomfortable	390	13.7%
Uncomfortable	471	16.5%
Neutral	1287	45.1%
Comfortable	488	17.1%
Very comfortable	220	7.7%
Are you aware of privacy settings and the importance of protecting personal information on social media?		
Yes	2699	94.5%

The impact of social media on participants’ mental health and well-being

Table 3 below presents participants’ responses to items that measured the impact of social media on participants’ mental health and well-being. The most commonly agreed-upon statements were that “they feel that excessive social media use has negatively affected their sleep patterns,” “they often sleep late in the night because of social media usage,” and “they check their social media account before they sleep in the night,” accounting for 71.0%, 66.1%, and 58.3%, respectively.

**Table 3:** The impact of social media on participants’ mental health and well-being

Variable	Frequency	Percentage
Do you feel that excessive social media use has negatively affected your sleep patterns? “Yes”	2029	71.0%
Do you often sleep late in the night because of social media usage? “Yes”	1888	66.1%
Do you check your social media account before you sleep in the night? “Always”	1664	58.3%
Do you feel like using your social media account in between your work or study? “Yes”	1638	57.4%
How supported do you feel by your social media connections when facing personal challenges? “Not supported at all or minimally supported”	1550	54.3%
Do you feel anxious without using your social media account just for two to three days? “Yes”	1428	50.0%
Do you think your behavior toward others has changed after using social media? “Yes”	1264	44.3%
Do you check your social media account just after waking up? “Always”	1176	41.2%
Do you feel pressure to portray a certain image or lifestyle on social media? “Yes, sometimes, or always”	1108	38.8%
Have you ever experienced symptoms of anxiety related to social media use? “Yes”	1080	37.8%
What do you feel for your memory power after using social media for years? “Decreased”	1077	37.7%
Do you have a constant desire of getting more likes and comments on your social media posts, and increasing your followers? “Yes”	1011	35.4%
Have you ever experienced symptoms of depression related to social media use? “Yes”	812	28.4%
How often do you encounter disturbing or distressing content on social media? “Often or very often”	800	28.0%
Do you feel that social media use has impacted your academic performance? “Yes, negatively”	749	26.2%
Have you ever been a victim of cyberbullying on social media? “Yes”	630	22.1%
How do you perceive your self-esteem when comparing yourself to others on social media? “Low or very low”	579	20.3%
How do you perceive the impact of social media on your overall life satisfaction? “Negative or very negative”	553	19.4%
How often do you feel fear of missing out (FOMO) when viewing others' social media posts? “Often or very often”	449	15.7%
How do you perceive your overall mental well-being? “Poor or very poor”	365	12.8%
How often do you compare your appearance with others on social media? “Often or very often”	354	12.4%

Predictors of being mentally affected due to social media

Table 4 below shows the findings of the binary logistic regression analysis. Overall, the study participants showed a low level of being mentally affected due to social media, with a mean score of 7.8 (SD: 3.9) out of 21, which is equal to 37.1%. Older participants (aged 16-24 years) were more likely to be mentally affected by social media compared to younger ones (aged 10-15 years) (p < 0.05). On the other hand, participants who were married, had a middle school education level, or lived in the northern, western, or eastern regions were less likely to be mentally affected by social media compared to others (p < 0.05).

**Table 4:** Predictors of being mentally affected due to social media * p < 0.05; ** p < 0.01; *** p < 0.001.

Variable	Odds ratio of being mentally affected (95% confidence interval)	p-value
Age group		
10-15 years (reference category)	1.00	
16-20 years	1.44 (1.09-1.92)	0.011*
21-24 years	1.41 (1.07-1.86)	0.014*
Gender		
Females (reference category)	1.00	
Males	1.03 (0.86-1.22)	0.774
Nationality		
Non-Saudi (reference category)	1.00	
Saudi	1.16 (0.89-1.51)	0.284
Marital status		
Single (reference category)	1.00	
Married	0.77 (0.64-0.93)	0.007**
Divorced	1.25 (0.69-2.25)	0.462
Widowed	1.54 (0.50-4.71)	0.453
Education		
Primary school or lower (reference category)	1.00	
Middle school	0.56 (0.31-1.00)	0.049*
High school	0.67 (0.41-1.11)	0.122
Diploma	0.69 (0.39-1.20)	0.190
University degree	0.80 (0.48-1.31)	0.374
Higher education	1.08 (0.53-2.19)	0.843
Employment		
Student (reference category)	1.00	
Employed	1.03 (0.84-1.25)	0.810
Unemployed	0.91 (0.74-1.12)	0.371
Region of residency		
Central region (reference category)	1.00	
Northern region	0.67 (0.52-0.86)	0.002**
Southern region	0.84 (0.66-1.05)	0.130
Western region	0.71 (0.57-0.88)	0.002**
Eastern region	0.58 (0.44-0.76)	<0.001***

## Discussion

Our study provided a comprehensive exploration of the multifaceted landscape of social media engagement among adolescents in Saudi Arabia, shedding light on the prevalent use of diverse platforms and its potential implications for mental well-being.

Twitter as a dominant platform: prevalence and impact

Twitter, a popular microblogging and social networking platform, allows users to create, tag, and share their content. In our study, we observed that Twitter emerged as the most commonly used social media platform among participants, with a notable prevalence of 30.3%. This aligns with broader trends indicating the platform's popularity among young individuals. Notably, Twitter users in Saudi Arabia hold the ninth position globally [20]. Based on prior survey data, 33% of individuals aged 13 to 17 years actively participate on Twitter [21]. This percentage increases to approximately 50% among 16 to 17-year-olds, as reported by another study [22].

The evolving dynamics of social media preferences among adolescents are highlighted in this observation, signifying its importance in understanding their digital behaviors and potential impacts on mental health. A prior study emphasizes a notable connection between active participation on Twitter and the emergence of depressive symptoms [23]. Interestingly, the study also found that consistent use of Facebook exhibited a protective influence against depressive symptoms [23]. This disparity underscores the various effects of different social media platforms on mental health, highlighting the need for further exploration into the associations between specific social media platforms and mental health outcomes.

Early initiation and device usage patterns: digital integration in adolescents' lives

The outcomes of our study, revealing that 52.5% of participants initiated their social media usage before the age of 10, align with data from previous studies. A prior study that examined the social networking activities of undergraduate students indicated that 61% of respondents across various countries joined their first social networking site before the age of 15 [24]. An average of 80% of children aged nine to 16 years, as highlighted by the European Union (EU) Kids Online survey covering 19 EU countries, engage in daily online activities using a smartphone [25]. This underscores the deeply integrated role of digital technologies and social media in children's day-to-day lives.

In Saudi Arabia, as reported in a prior report [26], the country's children lead in tablet device usage, with a notable 74% accessing such devices. Additionally, the report reveals that Saudi children emerge as the youngest demographic to possess their initial connected mobile device, typically obtaining one at the age of seven [26]. These statistics are reflective of the high internet penetration rate among children in Saudi Arabia.

These findings raise critical considerations regarding the timing of digital exposure and its potential implications for adolescent development and mental health. Earlier studies have indicated that children may be especially vulnerable to the adverse impacts of media use [27,28], which is also characterized as "developmental susceptibility" [29]. Notably, extensive use of social media in the teen ages has been demonstrated to influence personal well-being during adolescence, particularly among girls [28].

Despite the existence of age restrictions on social media platforms like Facebook and Snapchat as protective measures to safeguard children from online threats [30], there is a prevalent trend of underage users, including preteens and individuals under 13, actively participating in social networking sites, often without a comprehensive understanding of the potential consequences [31]. The early start of social media engagement among adolescents in Saudi Arabia is aligned with the global trend of fast integration of digital technology. This digital behavior emphasizes the need for comprehensive strategies to ensure responsible and well-informed use of social media among the younger age group. Addressing these challenges requires collaborative efforts from parents, policymakers, educators, and technology platforms to promote a supportive and healthy digital environment for adolescents in Saudi Arabia.

Escalating social media usage: implications and widespread concerns

In 2019, individuals in Saudi Arabia dedicated approximately three hours daily to social media applications [32]. Notably, our study revealed that around 37.7% of participants reported spending four to six hours per day on social media platforms, suggesting a potential increase in social media usage among the Saudi population. This observed trend aligns with a similar pattern reported in 2020, where children in the EU reported spending between two and 3.5 hours on the internet - a substantial rise compared to the initial EU Kids Online survey conducted in 2010 [33]. The substantial increase in daily social media use underscores the need for increased monitoring and awareness regarding the potential implications of extended online engagement.

Numerous previous studies have highlighted the adverse consequences of extended social media usage on mental health outcomes, encompassing depression, anxiety, and disruptions in sleep patterns among adolescents and young individuals [34]. The excessive use of social media, as highlighted in a Saudi study, has been identified as the origin of bullying. This research revealed that teenagers addicted to more than three hours of daily social media use often lack supervision [35]. An earlier study conducted among students at King Khalid University in Saudi Arabia concluded that in the student population, both income and the amount of time spent on social media were substantial predictors of depression [36]. Moreover, a different study indicated a noteworthy distinction in mental health outcomes between adolescents using social media for less than two hours and those exceeding this threshold [37]. Those spending more than two hours daily exhibited higher levels of depression, anxiety, and stress, indicating poorer mental health compared to their counterparts [37]. A separate investigation has shown that girls spending three hours or more daily on social media had a 29% engagement in self-harm [38]. The percentage increased to 31% for girls who spent five hours or more on social media [38], underscoring a concerning link between extended social media use and adverse mental health outcomes.

The acknowledged link between extended social media use and adverse mental health outcomes emphasizes the critical need for enhanced mental health support and resources, especially for the younger demographic. The implementation of strategies to decrease the time spent on social media is crucial. Thus, we can actively diminish potential risks to mental well-being among this vulnerable group.

Awareness and proactive measures: understanding the digital landscape

The findings of our study underscore a notable increase in awareness among study participants regarding mental health resources and support available through social media platforms, with the vast majority (85.2%) acknowledging their awareness of these resources. Moreover, a substantial proportion (77.4%) of participants reported actively attempting to reduce their social media usage for mental health reasons, highlighting a growing awareness of the potential impact of online interactions on well-being. Notably, a significant portion of participants (69.9%) were neutral or expressed comfort in seeking professional help for mental health issues related to social media use, suggesting a growing openness to addressing such concerns through formal channels. Furthermore, the vast majority (94.5%) indicated awareness of privacy settings and the importance of protecting personal information on social media, implying a heightened awareness of digital well-being practices. These collective findings reflect an encouraging trend toward increased awareness and proactive measures among individuals in navigating the mental health landscape within social media. This is attributed to the influence of digital connectivity, educational programs [39], and campaigns [39] promoting mental health awareness and support.

Sleep patterns and social media impact: recognizing associations and navigating solutions

In our study, most participants expressed concerns about the impact of social media on their sleep patterns, with three predominant statements notably agreeing. Specifically, 71.0% of respondents felt excessive social media use negatively affected their sleep patterns. This aligns with prior studies that have consistently linked increasing social media use to poor sleep patterns among adolescents [40-42]. The prevalence of this issue underscores a widespread recognition of the negative influence of social media on sleep quality.

The second widely agreed-upon statement, acknowledged by 66.1% of participants, is that they often sleep late at night because of social media usage. This finding resonates with broader research indicating that blue light emanating from smartphones and similar devices can interfere with children’s sleep quality by suppressing melatonin secretion [43-45]. Children face a heightened susceptibility to diminished mental well-being and heightened psychological distress, whether attributed to the influence of social media or the devices themselves. This underscores the importance of understanding and diminishing related potential impacts.

Furthermore, 58.3% of respondents indicated that they check their social media accounts before going to sleep at night. This behavior, which is prevalent among the study participants, may explain the agreement of most participants on the first and second statements. Multiple prior studies concluded an association between higher daily media use and an increased risk of sleep problems [40,41,46].

These emphasize the need for targeted interventions and educational initiatives to increase awareness of the potential adverse effects of social media on sleep, particularly among younger individuals. Sleep disturbances are recognized as one of the primary 10 warning signs of suicide among adolescents [47]. Besides, prior studies have indicated that sleep disturbances could be a significant risk factor for various mental health problems in adolescents [48-50]. Addressing disrupted sleep patterns may serve as a protective measure in preventing mental health issues, particularly depression [50,51]. The American Academy of Pediatrics has recommended restricting device use to a maximum of two hours per day [52].

Mental well-being and social media: exploring impacts and contributing factors

In our study, overall, the study participants showed a low level of being mentally affected due to social media, with a mean score of 7.8 (SD: 3.9) out of 21, which is equal to 37.1%. The low level of reported mental impact underscores the potential for positive experiences and outcomes associated with social media engagement among the study cohort. As mentioned before, social media platforms offer opportunities for benefits like self-expression [53].

Moreover, the lower level of mental impact from social media among adolescents in Saudi Arabia could be attributed to several factors. Cultural norms and values in Saudi Arabia [54] may play a pivotal role in promoting more positive and balanced patterns of social media use, ultimately contributing to a more beneficial impact on mental well-being. Additionally, strong family and community ties prevalent in Saudi society [55] might provide a supportive environment, easing the potential unfavorable effects of social media. Educational programs, awareness campaigns, and targeted interventions in the region [38] also play a crucial role in encouraging beneficial online habits among adolescents, leading to a lower overall mental impact.

Demographic factors: exploring associations with mental impact

In our study, an age-related susceptibility to the mental impact of social media emerged, with older participants (aged 16-24 years) exhibiting a higher likelihood of being affected compared to their younger ones (aged 10-15 years) (p < 0.05). Conversely, participants who were married, had a middle school education level, or lived in the northern, western, or eastern regions were less likely to be mentally affected by social media compared to others (p < 0.05). These findings highlight several significant associations between demographic factors and the mental impact of social media in our study population.

Consistent with our results, research has highlighted variations in the occurrence of depression and anxiety across different age groups. Specifically, younger adults (16 to 29 years) exhibited a higher susceptibility to depression and severe anxiety compared to their older counterparts [56]. Additionally, a national survey in Australia revealed that individuals in the 16-24 years age range had the highest prevalence (26%) of experiencing anxiety, affective disorders, or substance use disorders within the past years, exceeding rates in other age groups [57]. This emphasizes the significance of age as a factor in understanding the prevalence of mental health issues, aligning with our study's focus on age-related differences in the mental impact of social media.

The variations in the likelihood of adolescents in Saudi Arabia being mentally affected by social media based on marital status, education level, and geographic region may be rooted in Saudi Arabia's cultural and societal dynamics, as mentioned earlier. Marriage, particularly when characterized by happiness, is linked to enhanced mental well-being [58]. Moreover, married individuals might find support beneficial in easing the potential negative impact of social media on mental well-being [59]. Those with a middle school education may exhibit greater awareness of the effects of social media, potentially adopting more balanced online habits. Geographic regions may contribute to distinct social norms and community dynamics, shaping adolescents' perceptions and interactions with social media. Collectively, these factors emphasize the influence of cultural, educational, and regional contexts on the mental impact of social media among adolescents in Saudi Arabia.

Individuals with advanced educational backgrounds may encounter a more significant psychological influence from social media as a result of heightened exposure to academic stress, performance demands, and competition. Individuals with a higher level of education are more likely to interact with online content in a more profound manner, which could potentially intensify the impact of social comparison and cyberbullying. Moreover, their superior cognitive capabilities may result in increased self-awareness, rendering them more vulnerable to mental health issues.

It is imperative to comprehend the impact of social media utilization on the mental well-being of adolescents in Saudi Arabia, owing to the substantial youth population, swift technological advancements, and cultural complexities. It is imperative to customize interventions according to localized research to cultivate a more salubrious digital environment and advance well-being within this constantly developing societal framework. This study is among the first few studies to examine the mental effect of social media on adolescents in Saudi Arabia.

This study has limitations. The cross-sectional study design restricted the ability to examine causality among the study variables. In surveys, response bias and recall bias are two common limitations that can compromise the accuracy of responses. Response bias emerges when participants provide inaccurate or partial information due to influences such as social desirability bias, which refers to the inclination to provide affirmative or socially acceptable responses. In the same way, recall bias pertains to the inclination of participants to provide biased responses by inaccurately recollecting events or information.

Additional research on the effects of social media on the mental well-being of Saudi Arabian adolescents should focus on long-term patterns, cultural factors, and the effectiveness of interventions. Policymakers ought to contemplate the adoption of protocols for conscientious utilization of social media, specifically targeting the resolution of emerging barriers.

## Conclusions

The study participants showed low levels of being mentally affected due to social media. Older participants (aged 16-24 years) were more prone to be mentally affected due to social media. Parents and caregivers have the ability to decrease the amount of time spent on social media by establishing explicit time constraints, promoting transparent communication, exemplifying good online conduct, and endorsing a variety of offline activities. Building trust and implementing parental restrictions are key factors in adopting a well-rounded approach that fosters healthy digital habits in children. It is not the primary responsibility of parents and caregivers to mitigate the potential risks of social media. Promoting the establishment of technology-free areas and encouraging adolescents to cultivate face-to-face friendships could be a good strategy that decision-makers should endorse.
